# Consecutive Low Doses of Cyclosporine A Induce Pro-Inflammatory Cytokines and Accelerate Allograft Skin Rejection

**DOI:** 10.3390/molecules16053969

**Published:** 2011-05-11

**Authors:** Roberto López-Flores, Rafael Bojalil, José C. Benítez, Yadira Ledesma-Soto, César A. Terrazas, Miriam Rodríguez-Sosa, Luis I. Terrazas

**Affiliations:** 1 Carrera de Medicina, Facultad de Estudios Superiores Iztacala, Universidad Nacional Autónoma de México (UNAM), Av. de los Barrios Num 1, Los Reyes Iztacala, Tlalnepantla, Estado de México, Mexico; 2 Departamento de Inmunología, Instituto Nacional de Cardiología, Juan Badiano Num1, Tlalpan, México, D.F., Mexico; 3 Unidad de Biomedicina, Facultad de Estudios Superiores Iztacala, Universidad Nacional Autónoma de México (UNAM), Av. de los Barrios Num 1, Los Reyes Iztacala, Tlalnepantla, Estado de México, Mexico

**Keywords:** cyclosporine A, Treg, graft rejection

## Abstract

Cyclosporine A (CsA) is a fungus-derived molecule with potent immunosuppressive activity that has been largely used to downregulate cell-mediated immune responses during transplantation. However, previous data have indicated that CsA shows immunomodulatory activity that relays on the antigen concentration and the dose of CsA used. To test the hypothesis that minimal doses of CsA may show different outcomes on grafts, we used an experimental model for skin transplants in mice. ICR outbred mice received skin allografts and were either treated daily with different doses of CsA or left untreated. Untreated mice showed allograft rejection within 14 days, with graft necrosis, infiltration of neutrophils and macrophages and displayed high percentages of CD8^+^ T cells in the spleens, which were associated with high serum levels of IL-12, IFN-γ and TNF-α. As expected, mice treated with therapeutic doses of CsA (15 mg/kg) did not show allograft rejection within the follow-up period of 30 days and displayed the lowest levels of IL-12, IFN-γ and TNF-α as well as a reduction in CD8^+^ lymphocytes. In contrast, mice treated with consecutive minimal doses of CsA (5 × 10^−55^ mg/kg) displayed an acute graft rejection as early as one to five days after skin allograft; they also displayed necrosis and strong inflammatory infiltration that was associated with high levels of IL-12, IFN-γ and TNF-α. Moreover, the CD4^+^ CD25^hi^FoxP3^+^ subpopulation of cells in the spleens of these mice was significantly inhibited compared with animals that received the therapeutic treatment of CsA and those treated with placebo. Our data suggest that consecutive, minimal doses of CsA may affect Treg cells and may stimulate innate immunity.

## 1. Introduction

When allograft tissues differ from the host at class I and class II loci, CD8+ T cells are activated through recognition of the graft alloantigens. The CD8+ T cells recognize foreign MHC class I molecules expressed on all donor graft cells. Differentiation of cytotoxic T lymphocytes (CTLs) is generally regulated by CD4^+^ T cells, which are stimulated by antigen-presenting cell (APC) class II molecules in the graft. Indeed, tissue allografts that induce strong rejection reactions present APC class II molecules. Recently, it has been observed that CD8^+^ T cells probably depend on the same APC as CD4^+^ T cells. APCs are the most important stimulators of the immune response in the graft interstice. The key characteristic of APCs is the presence of co-stimulatory molecules, which allow the activation of CD8^+^ and CD4^+^ T cells [1,2,3,4,5,6,7,8,9].

At present, the most common immune suppressor used during transplants is cyclosporine A (CsA). In contrast to other cytotoxic immune suppressors, CsA does not kill immune effector cells but rather selectively inhibits their proliferative activation and expansion, mainly by interfering with IL-2 synthesis. However, this unique suppressive activity of CsA was first challenged by Bretscher and Havele [10], who found that CsA used in low doses together with a specific antigen could bias a humoral immune response toward a cell-mediated immune response or *vice versa*, depending on the concentrations of the inducing antigen and CsA [10]. In recent years, a novel view of CsA has emerged; several groups of researchers have found diverse activities of CsA, such as inhibition of inflammatory reactions and regulation of the activity and expression of inducible Nitric Oxide Synthase (iNOS), suggesting the use of an old drug in new or alternative ways to treat different pathologies, such as experimentally-induced spinal cord injury [11].

Thus, the interesting immunoregulatory properties of cyclosporine A described by Bretscher and Havele, in addition to the recent activities reported for CsA, motivated us to use an experimental model of skin allograft in mice to test the hypothesis that minimal doses of CsA may affect the immune response. In an attempt to dissect the mechanisms that lead to immune modulation in response to CsA treatment, we examined the role of consecutive low doses of CsA in mice receiving an allograft skin transplant. Our data show that this kind of treatment significantly accelerates graft rejection; whereas an important APC population such as dendritic cells (DCs) were unaffected by low dose of CsA, another important immuno-regulatory population such as Treg cells were significantly altered along with an increased detection of pro-inflammatory cytokines. Thus, CsA appears to play a more sensitive role in modulating the immune response. 

## 2. Results and Discussion

### 2.1. Treatment with Consecutive Low Doses of CsA Accelerates Skin Allograft Rejection

**Figure 1:**
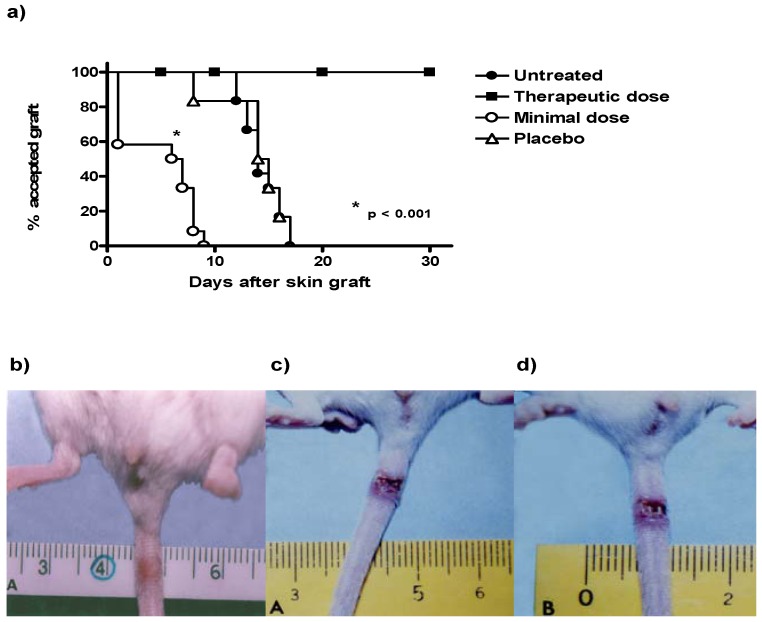
Time course of allograft survival in ICR mice after treatment with consecutive low dose of CsA. (**a**) Survival graft: Animals were grafted in the tail and survival of grafts was monitored for 30 days. The data are representative of two independent experiments (n = 10–12 mice per group). * P < 0.01. (**b–d**) Photographs of tails showing the rejection graft, therapeutic dose of CsA, placebo and consecutive low-dose of CsA, respectively.

As expected, mice with skin allografts that did not receive any treatment rejected the transplanted skin 14 days after surgery, like the mice that received the placebo (Figure 1a). In contrast, mice that received treatment with minimal doses of CsA (CsA Low dose) significantly accelerated the rejection time of the allograft, reducing the time required for skin rejection by almost threefold from 14 days in untreated animals to 5 days in mice receiving minimal doses of CsA (Figure 1a and d). The animals treated with therapeutic doses of CsA did not reject the transplant during the 30 days of follow-up (Figure 1a). Figure 1b shows the tail of a mouse with an accepted skin allograft, whereas Figure 1c–d shows mice with skin allograft rejection, without treatment and treated with consecutive low dose of CsA, respectively.

### 2.2. Histopathology Analysis of Skin Allograft Shows Acute Rejection in Mice Given Low Doses of CsA

**Figure 2:**
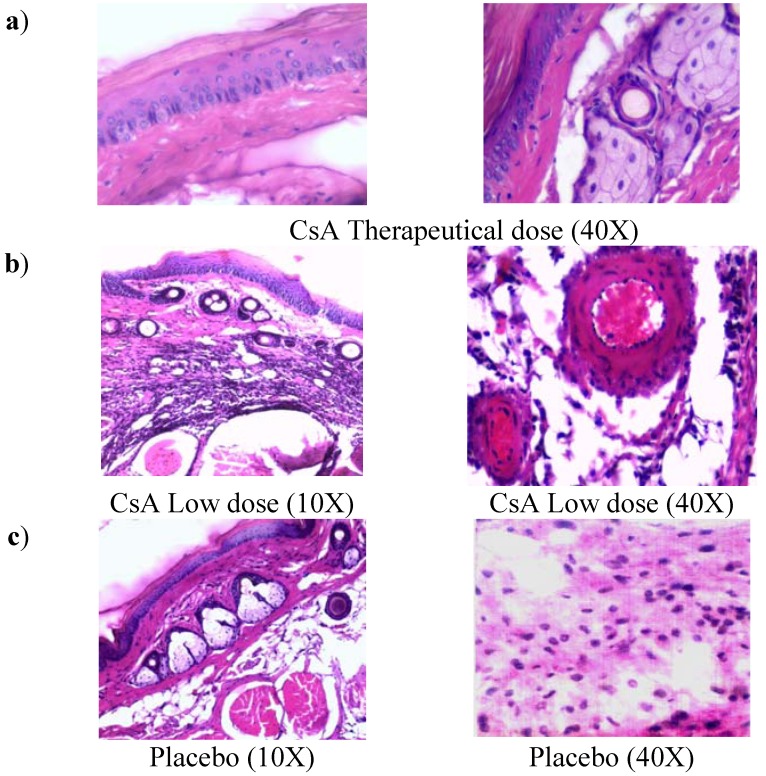
Histology of grafted skin. **a**) Skin histology of therapeutic CsA-treated group, where graft was incorporated in the host tissues, the dermis presents abundant phagocytic cells but not necrosis, some intact sebaceous glands can be observed. **b**) Skin histology of grafted mice under consecutive low dose of CsA-treatment, where epidermis and dermis were found necrotic. Cell infiltrate with abundant neutrophils together with atrophic muscular beams were found. c) Image of grafted skin from mice treated with placebo. All photos were taken 7 days after skin allograft.

Control mice with transplants but no treatment showed some inflammatory necrosis and little infiltration of macrophages and neutrophils at day 14 after the graft (Figure 2a). In contrast, treatment with low doses of CsA induced a strong rejection reaction on the first days of treatment against the implanted dermis and epidermis, in which a severe neutrophil infiltration can be observed, with some macrophages. There were also some necrotic areas around a couple of arteries (Figure 2b, 40X) and in some muscle fibers. In contrast with all other groups, the graft-receiving host dermis showed intense macrophage and neutrophil phagocytic activity against muscular and nerve tissue, which may stimulate degenerative processes in the epidermis and glands. In contrast, allografts in mice that received a therapeutic treatment of CsA did not show the above-mentioned reactions (data not shown). In mice given therapeutic doses of CsA, images of a graft that was incorporated into host tissues showed a region of skin alongside the graft with intact sebaceous gland (data not shown and Figure 2b). The mice treated with the placebo formulation of therapeutic and minimal low doses of CsA solvents induced transplant rejection with characteristics similar to the control group with transplant and no treatment (placebo).

### 2.3. Mice Receiving Consecutive Minimal Doses of CsA Display a Pro-inflammatory Cytokine Profile

**Figure 3:**
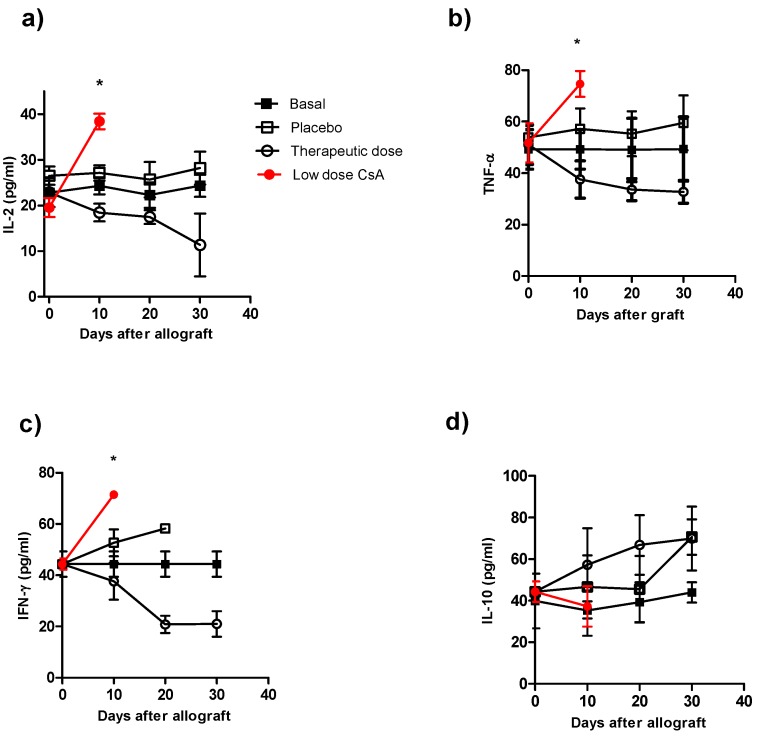
Kinetics of cytokine production during course of allograft in ICR mice receiving different treatment of CsA. Individual mice were bleeding at different times after skin allograft, and sera were processed for cytokine detection by ELISA **a**) IL-2 detection, **b**) TNF-α detection, **c**) IFN-γ detection and **d**) IL-10 detection in sera of from animals receiving allografts and CsA treatment. The data are the mean of two independent experiments at each time point. An asterisk indicates statistically significant (*P < 0.05) differences between the groups receiving CsA with respect to basal and placebo.

To correlate our findings on graft rejection with the cytokine profile in the different groups, we analyzed the serum levels of IL-2, IL-12, IFN-γ, TNF-α and IL-10. The control groups (animals with transplants without treatment and the control group plus placebo), which displayed normal rejection conditions, produced higher levels of IL-2 (Figure 3a), TNF-α (Figure 3b) and IFN-γ (Figure 3c) early after skin allograft than the naïve group; however, these levels were lower than those in mice that received the minimal dose of CsA. This latter group produced the highest levels of pro-inflammatory cytokines (Figure 3a–c). In contrast, the production of IL-2, TNF-α and IFN-γ was lower in mice given the therapeutic CsA treatment (Figure 3a–c). Interestingly, no differences in IL-10 production were detected among the groups (Figure 3d). The animals that received the therapeutic doses showed the lowest levels of IL-2, IFN-γ and TNF-α compared with all of the other groups but similar levels of IL-10 (Figure 3a–d). The cytokine levels in mice receiving low doses of CsA were not followed after 10 days because by that time 100% of these mice displayed graft-rejection. 

**Figure 4:**
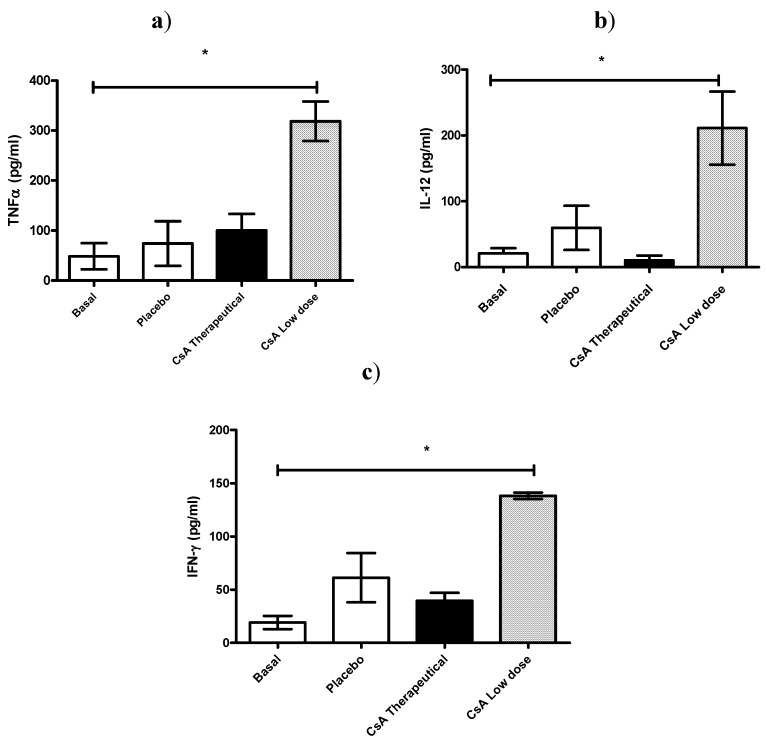
Effect of CsA on pro-inflammatory cytokine levels on sera early after rejection. (a–c) ICR mice, 6 mice per group, received placebo or 15 mg/kg of CsA (therapeutic dose) or 5 × 10^−55^ mg/kg of CsA (low dose) daily since the time of skin transplantation. Seven days later cytokines (TNF-α, IFN-γ and IL-12) were analyzed by ELISA.

In another set of experiments, all mice were killed 7 days after skin allograft, and sera was analyzed for IL-12, TNF-α and IFN-γ production. As can be observed in Figure 4 all these pro-inflammatory cytokines were significantly elevated in mice receiving daily low dose of CsA and which already had rejected the graft (Figure 4 a–c).

The contradictory effects of very low and consecutive doses of CsA can be explained in part by the early observations of Bretscher and Havele [10], who found that incremental doses of CsA to mice reconstituted with 5 × 10^6^ spleen cells progressively inhibited DTH responses. The cell-mediated response occurred first, but was subsequently overtaken by the humoral response, as demonstrated when chicken erythrocytes were used as the antigen [10]. However, when incremental CsA doses were given to mice reconstituted with 35 × 10^6^ spleen cells, there was an initial antibody reaction that was gradually inhibited while the DTH reaction simultaneously increased, which was also shown using chicken erythrocytes as the antigen [10]. 

These findings [10] may suggest different immunological targets depending of the dose of CsA used. In fact, a recent report has indicated that CsA reduced IL-2 production and DTH reactions in BALB/c mice, but not in B6D2F1 mice, which suggest that *in vivo* there are different responses to similar doses of CsA [12]. Here we observed that mice treated with consecutive minimal doses of CsA exhibited spontaneous premature rejection of the transplants after an average of five days, which was associated with an increase in the levels of IL-2, IL-12, IFN-γ and TNF-α but without affecting IL-10. 

It is well-known that CsA downregulates transcription of the IL-2 gene. As a result the main target of this agent is the T helper cell. In contrast, CsA exhibited minimal toxicity on preformed cytotoxic/suppressor T cells, B lymphocytes, granulocytes and macrophages [13,14,15,16,17,18]. This drug is a very potent and relatively selective inhibitor of T cell responses, particularly those responsible for graft rejections [19,20]. However, relatively recent observations also suggest that CsA may act differentially *in vivo* affecting distinct cell populations, for example it has been demonstrated that the same dose of CsA can suppress *in vivo* both NKT and dendritic cell maturation, but not NK cells in response to alpha-galactosylceramide [21]. 

### 2.4. Minimal Doses of CsA Increase the CD4^+^ T Cell Subpopulation

**Figure 5:**
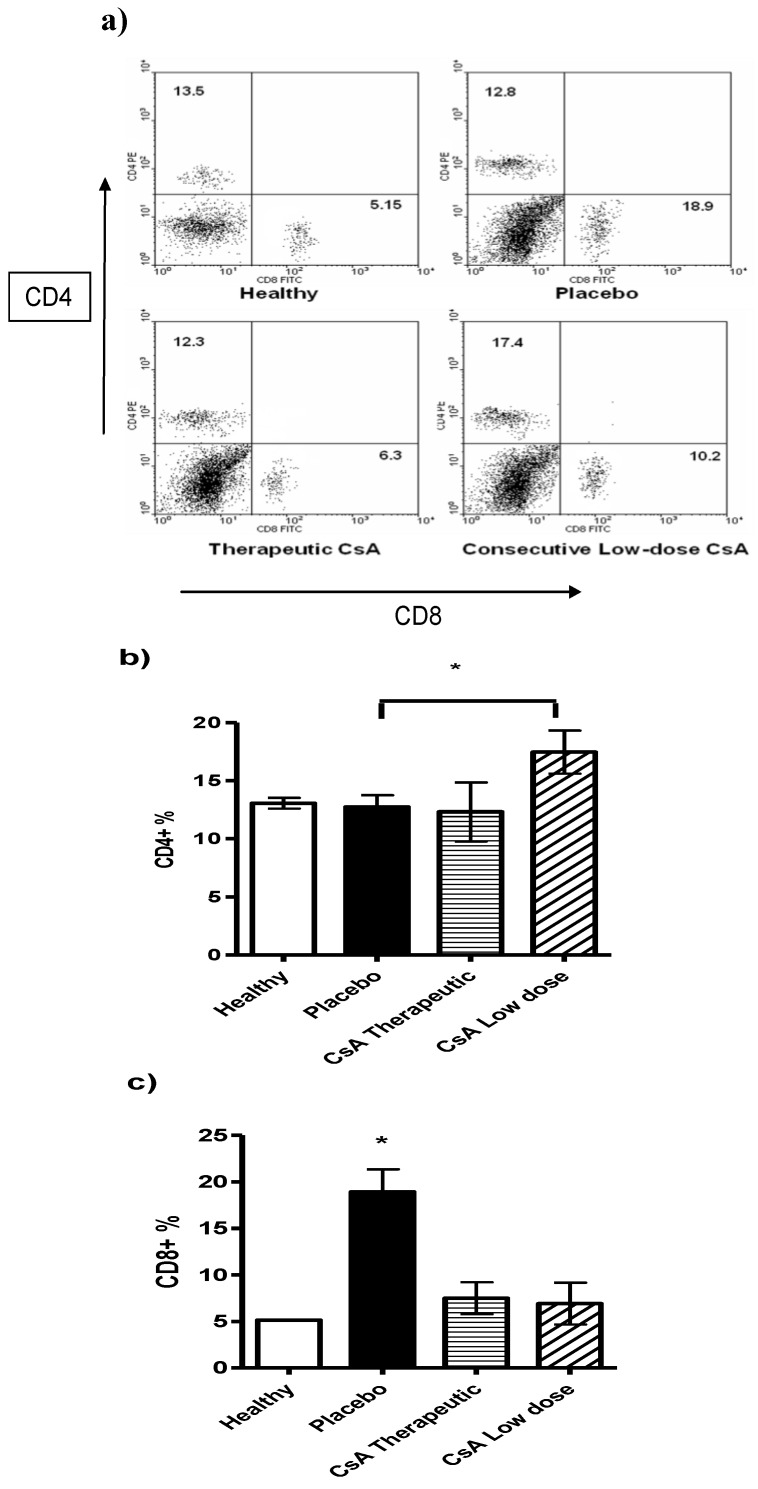
Flow cytometry analysis of spleen cells from mice grafted and differentially treated with CsA. Spleen cells were obtained after graft rejection or after finished the follow-up period of time. CD4 and CD8 positive cells were analyzed. **a**) Representative dot plot of spleen cells from naïve mice, placebo-treated mice, therapeutic dose of CsA and consecutive low dose of CsA. Numbers indicate mean percentage of positive cells. **b**) Average of percentage of CD4^+^ cells and c) percentage of CD8^+^ cells. Bars show average ± SD, n = 4 mice per group; * P < 0.05.

Following the above idea we analyzed different lymphocytic populations such as CD8^+^ and CD4^+ ^T cells in the spleens of mice with skin allograft that received the different treatments. Mice with skin allograft that received the placebo displayed a significant increase in the percentage of the CD8^+^ T-cell subpopulation compared with naïve mice (Figure 5). In contrast, animals given minimal doses of CsA showed significantly higher percentages of CD4^+^ T cells in the spleens than mice given the therapeutic doses or the placebo. Interestingly, the placebo group also displayed more CD8^+^ T cells than the therapeutic treatment group, but the difference was not statistically significant. In contrast, mice that received high doses of CsA had significantly less CD8^+^ T cells than those treated with the placebo (Figure 5c).

### 2.5. CsA May Affect Other Immune Cell Populations

**Figure 6:**
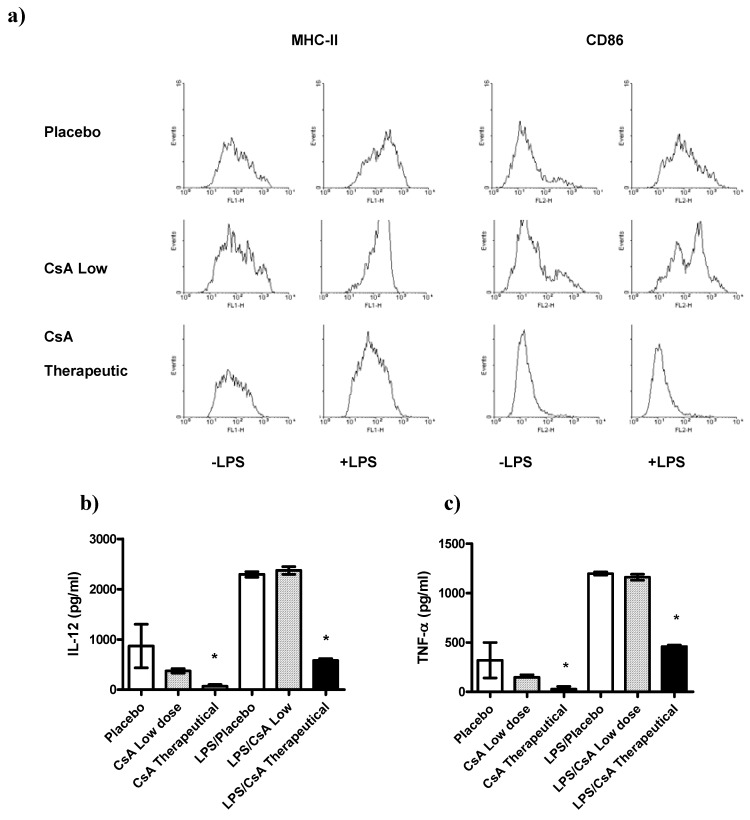
Differential effect of CsA doses on dendritic cells (DC) maturation and activation. DCs were developed from bone marrow of healthy mice, and culture with GM-CSF for 5 days, then DCs were stimulated or not with LPS 1 μg/mL in presence of therapeutic or low dose of CsA or placebo. **a**) Low dose of CsA do not alter DC maturation, but therapeutic dose of CsA significantly inhibits DC expression of MHC-II and CD86. **b-c**) Twenty four hours later supernatants were collected and analyzed for IL-12 and TNF-α production by ELISA. * p < 0.05 compared with placebo. Data represent 2 independent experiments.

Recently it has been demonstrated that other cell types besides T cells can be affected by CsA [21,22,23]. In order to know whether or not other important regulatory cells were affected by the low doses of CsA treatment we analyzed whether exposure of DCs to therapeutic or low doses of CsA *in vitro* may alter the pro-inflammatory response and maturation markers of those cells to LPS-mediated stimuli. The maturation markers (MHC-II and CD86) as well as the production of IL-12, and TNF-α two key cytokines associated with inflammatory responses, were assayed. We detected that DCs exposed to therapeutic dose of CsA inhibited the expression of the maturation markers MHC-II and CD86 (Figure 6a), in contrast, DCs exposed to low dose of CsA displayed a typical maturation, similar to that obtained with placebo (Figure 6a). In line with these observations, exposure of DCs to placebo or CsA low dose for 24 h did not induce significant secretion of IL-12 or TNF-α compared with unstimulated DCs (Figure 6b–c). In contrast, DCs exposure to therapeutic CsA dose showed lower levels of these two inflammatory cytokines (Figure 6b–c). As expected, we found that BMDCs stimulated with LPS (1 μg/mL), induced high production of the pro-inflammatory cytokines IL-12 and TNF-α (Figure 6b–c). We then analyzed whether acute exposure of DCs to different doses of CsA may influence the response to LPS-mediated stimuli. BMDCs were exposed to CsA and immediately stimulated with LPS for 24 h. Exposure to therapeutic dose of CsA down-regulated the production of both pro-inflammatory cytokines tested (Figure 6b–c). Interestingly, CsA in low dose did not alter the production of both IL-12 and TNF-α (Figure 6b–c). 

DCs are also important in modulating early responses, here we found that DCs were highly sensitive to therapeutical dose of CsA given that DCs exposed *in vitro* to such amount of CsA were unable to maturate in response to LPS and also showed a significant low production of inflammatory cytokines, in contrast, exposure to low dose of CsA did not affect either maturation or cytokine production of DCs. These data are in line with those reported for the inhibition of DC maturation *in vivo* using high doses of CsA [24]. We also analyzed other cells such as NK cells which were unaltered (data not shown). 

**Figure 7:**
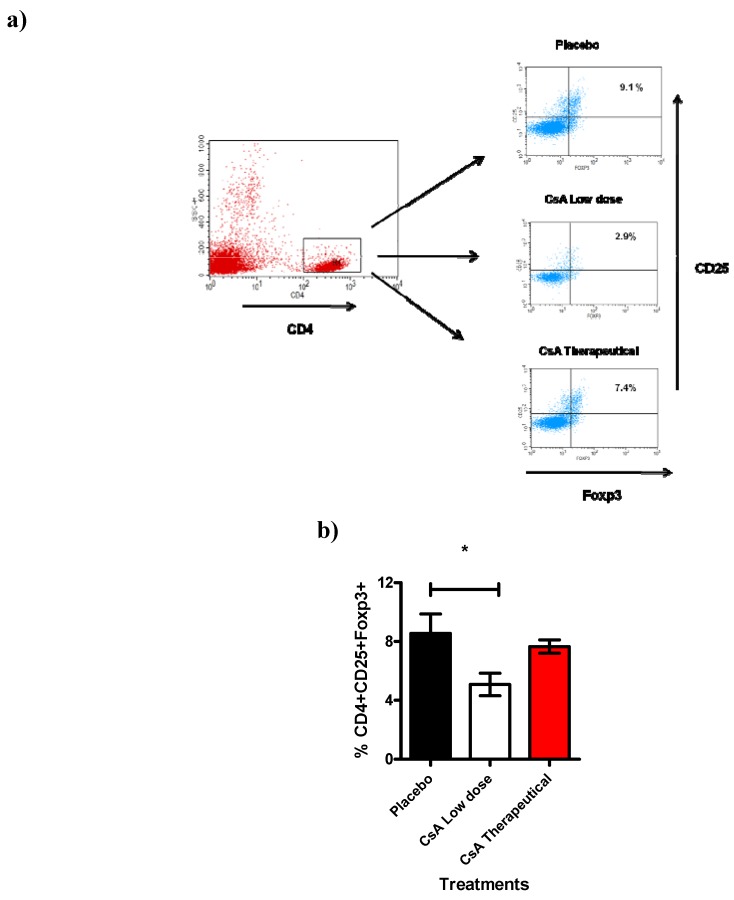
Treg cell population is affected by Low dose of CsA. This figure shows the flow cytometric analysis of T regs population from the spleen. **a**) Representative dotplot indicating the percentage of CD25^+^Foxp3^+^ cells obtained from the gate of CD4^+^ cells from spleen of grafted mice under different treatments (7 days after graft). **b**) Analysis of total Tregs, data are representative of two independent experiments, bars indicate standard deviation, n = 4 mice per group. *P < 0.02 according to one way ANOVA.

Next we analyzed another cell population also involved in transplantation such as the CD4^+^CD25^+^ T reg cells [26]. Here we studied the total spleen cells 7 days after allograft. Spleens were removed and analyzed for the expression of CD4^+^CD25^hi^Foxp3^+ ^cells. Interestingly, we found a significant reduction (approx. 30 to 40%) of cells expressing CD4^+^CD25^hi^Foxp3^+ ^in mice that were treated with consecutive low dose of CsA during 7 days and that had already rejected the skin allograft (Figure 7), treatment with placebo or therapeutic dose of CsA were similar and displayed higher percentages of Tregs. This intriguing result may support our data regarding to higher levels of inflammatory cytokines as well as the faster graft rejection, probably because the limited regulatory activity of the remaining Treg cells.

In different experimental transplant models, classical alloreactive CD4^+^ or CD8^+^ T cells along with specialized alloantibodies regulate allograft rejection reactions after transplantation. In addition, rejection may be inhibited by anti-CD4 or anti-CD8 antibodies or accelerated by depletion of CD4^+^CD25^+^ cells. These different immunoeffectors result in graft rejection through various mechanisms: (a) Alloreactive T cells may recruit and activate macrophages, initiating graft injury through a delayed-type hypersensitivity (DTH) reaction; (b) alloreactive T cytotoxic cells directly provoke lysis of endothelial and parenchymal cells of the graft; (c) antibodies attack the endothelial tissue, activate the adaptive immune system and cause injuries in the blood vessels of the graft and (d) the absence or lower percentages of CD4^+^CD25^+^ Foxp3^+^ T regs may allow a more rapid and strong pro-inflammatory response that active and recruit innate cells such as neutrophils and macrophages [6,7,8,9]. In this study we found an association between the lower percentages of Tregs with faster graft rejection in mice receiving consecutive low dose of CsA which displayed stronger responses and 100% of allografts were rejected in less than ten days. This effect was also associated with neutrophil and macrophage accumulation and with higher levels of IL-2, IFN-γ and TNF-α.

The reported activation of the cell-mediated immune response by minimal doses of CsA may induce acute cellular rejection [10], but this kind of rejection is characterized by necrosis of parenchymal cells and is often associated with infiltration of lymphocytes and macrophages. Here we observed mainly a severe neutrophil infiltration in the skin that was promptly rejected in mice treated with consecutive low dose of CsA, such important difference may involve a distinct mechanism of rejection. 

Even thought that our results are surprising because the immunosuppressive effects of CsA have been attributed to the prevention of IL-2 transcription [27], they are in line with a recent report by Miroux et al who found *in vitro* that a low dose of CsA can inhibit T regulatory cells activity whereas a high dose had no effect in this T cell population [23], interestingly here we observed a similar phenomena but *in vivo*. It is well known that Treg cells can mediate the induction and maintenance of immunological self-tolerance as well as transplant tolerance, their inhibition by CsA might promote graft rejection [28]. Thus, further research is guaranteed to evaluate whether or not consecutive low dose of CsA *in vivo* can affect the activities of these subpopulations. Moreover, a recent report has indicated that *in vivo* CsA reduced IL-2 production and DTH reactions in BALB/c mice but not in B6D2F1 mice, which suggest that *in vivo* there are different responses to similar doses of CsA [12]. Moreover, other drug such as cyclophosphamide has been used at low doses in immunotherapy for mesothelioma tumors where it was found that low doses of cyclophosphamide induced beneficial effects by preventing the increase of Treg cells [25]. Consequently, minimal and consecutive doses of CsA deserve further and deeper research in order to, in a near future, may considered to stimulate innate immune responses in the treatment of certain illnesses, such as miliary tuberculosis, leprosy and some parasitic diseases [29], given that CsA is already an authorized drug for human use. In fact, the puzzling aspect of the effect of different doses on the targets or on the complete individual is getting bigger relevance with other drugs such as rapamycin which depending of the dosage it can influence different pathways [22] .

## 3. Experimental

### 3.1. Animals

Ten- to twelve-week-old female mice of the outbred ICR strain were purchased from Harlan Laboratories (Mexico) and used in all of the experiments. All mice were housed under specific pathogen-free conditions at the FES-Iztacala, UNAM animal facility in accordance with institutional and national guidelines.

### 3.2. Surgery Procedures

Two mice at a time were anesthetized using intraperitoneal injection with pentobarbital sodium (0.60 mg per gram of body weight). A small portion of skin (5 × 5 mm) was removed from the ventral part of the tail section close to the body and exchanged between the mice. The transplant was then covered with collodion scarring liquid, and a glass protective cover was fastened with micropore surgical tape. All materials used were sterile, and all transplants were performed in the surgical room at the animal research facility.

### 3.3. Cyclosporine A Preparation

CsA (Lemery S.A., México D.F.) was prepared as follows: an ampoule of CsA (50 mg in a 1-mL volume of castor oil (650 mg) and 35% alcohol was added to a 10-mL amber glass bottle with a screw-top lid. Then, polyethylene glycol (1 mL), physiological saline solution (1 mL) and ethanol (0.33 mL) were added, resulting in 50 mg of cyclosporine A in a total volume of 3.33 mL. At this dilution, 0.1 mL was equal to 1.5 mg of cyclosporine A (dose of 15 mg/kg/day). The minimal dose of cyclosporine A was prepared with an ampule of 50 mg cyclosporine A in 1 mL polyethylene glycol (JT Baker) and 96° alcohol, according to the following dilution series:

1^st^ – 9 mL polyethylene glycol + 1 ampule of 50 mg CsA in 1 mL.
2^nd^ – 9 mL polyethylene glycol + 1 mL of the first solution.
3^rd^ – 9 mL polyethylene glycol + 1 mL of the second solution.
4^th^ – 9 mL polyethylene glycol + 1 mL of the third solution.
5^th^ – 99 mL ethanol + 1 mL of the fourth solution.
6^th^ – 99 mL ethanol + 1 mL of the fifth solution.

Subsequent dilutions were prepared following this procedure, adding ethanol (99 mL) to the former solution (1 mL) until the 30^th^ serial dilution. Each solution was strongly agitated before the following dilution was prepared. To prepare the injectable solution, physiological saline solution was used instead of alcohol for the 30^th^ dilution, which was not agitated, only mixed. The 30^th^ dilution solution had a concentration of 5 × 10^−55^ mg/mL of CsA and was adjusted to inject 5 × 10^−55^ mg/kg.

### 3.4. Treatments

A total of 94 ICR strain female mice weighing between 30 and 35 g were used in this study. Of the 94 mice, 72 were divided into three groups of 24 mice each. From each group, 12 mice were kept alive until transplant rejection, and the other 12 mice were sacrificed for histopathologic evaluation of the skin transplant. The remaining 22 mice were divided into two control groups, leading to a total of five groups. Group 1 (naïve) comprised 10 mice that did not undergo transplants or treatment and were maintained to determine the basal values of the cytokines of interest. Group 2 (placebo) comprised 12 mice that were treated with placebo formulations of CsA solvents. Six mice were treated with therapeutic doses, and the other six were treated with minimal doses. Group 3 (control) comprised 24 mice with allografts but without treatment. It was divided into two subgroups of 12 mice each. Subgroup A rejected the transplant after an average of 14 days. Each day, one mouse from subgroup B was sacrificed for histopathologic analysis of the transplanted skin region. Group 4 (minimal dose) comprised 24 mice with allografts that were subcutaneously injected with minimal doses (0.1 mL) of CsA (5 × 10^−55^ mg/mL) daily. This group was divided into two subgroups of 12 mice each. Subgroup A rejected the transplant spontaneously after five days, on average. Each day, one mouse from subgroup B was sacrificed for histopathologic analysis of the transplanted region of the skin. Group 5 (therapeutic dose) comprised 24 mice with skin allografts that were treated with therapeutic doses of CsA (15 mg/kg) injected subcutaneously every day. It was divided into two subgroups of 12 mice each. Subgroup A did not reject the transplant during the month of observation. Every third day, one mouse from subgroup B was sacrificed for histopathologic analysis.

### 3.5. Histological Analysis

The inserted skin grafts were fixed in 10% formaldehyde and embedded in paraffin according to conventional methods. Histologic sections of 6 µm were prepared and stained with hematoxylin and eosin. Images were captured using the Nikon Labophot II.

### 3.6. Determination of Cytokine Concentrations

The levels of IL-2, IL-10, IL-12, IFN-γ and TNF-αwere determined in the group of mice without transplants and treatment to identify basal concentrations. In the control and therapeutic treatment group, IL-2, IL-10, IL-12, IFN-γ and TNF-α levels were determined four times: once before the transplant and three times after the transplant every ten days. In the minimal treatment group, cytokine concentration evaluation was only performed twice: once before the transplant and once after ten days because the skin transplant was rejected after an average of five days. In some experiments such determinations were made 7 days after skin transplantation. All cytokines were detected using a sandwich ELISA in accordance with the manufacturer’s instructions (Peprotech, Mexico).

### 3.7. Flow Cytometry

Total spleen cells were isolated from mice of all groups, washed, incubated with anti-CD16/CD32 (Biolegend, San Diego, CA. USA) for 30 min at 4 °C and then suspended in 3% BSA-PBS. Phenotypic analysis of spleen cells was conducted using direct immunofluorescence staining and FACS analysis. Cells (1 × 10^6 ^/mL)were incubated with FITC- and/or PE-labeled anti-CD4 and -CD8 antibodies (Biolegend) at 4 °C for 30 min. Surface expression of DC maturation markers was analyzed using multicolor flow cytometry. DCs (either untreated or stimulated for 24 h with LPS, LPS+placebo, CsA therapeutic dose or CsA low dose or placebo alone) were harvested, washed and suspended in cold PBS containing 5% FCS and 0.05% NaN_3_. F_c_ receptors were blocked with anti-mouse CD16/CD32 for 20 min at 4 °C. Cells were washed and triple stained with an APC-conjugated antibody against CD11c, FITC-conjugated monoclonal antibodies against CD86, or Phycoeritrin-conjugated antibodies against MHC-II [Major Histocompatibility Complex] (all antibodies from Biolegend, USA). In some experiments we evaluated the percentages of T regulatory cells using the T-regs kit from Biolegend, all directions suggested by the manufacturer were followed. After incubation, cells were washed several times in buffer, fixed in 1% paraformaldehyde (Sigma-Aldrich) and stored at 4 °C in the dark before analysis using FACS Calibur and CellQuest software (Becton Dickinson Franklin Lakes, NJ, USA). *Statistical analysis*. Data are expressed as the means ± SD. The statistical significance of differences in mean values was determined using the Student’s *t*-test or One way ANOVA. Survival data on grafts are presented as Kaplan-Meier survival curves and were analyzed using the Log-rank test. Differences of P < 0.05 were considered significant.

## 4. Conclusions

CsA is an immunosuppressive agent that has been widely used to inhibit exacerbated immune responses during transplant rejection [9] and autoimmune diseases [30]. However, due to its mechanism of action (inhibition of calcineurin activity), CsA can also exert beneficial effects after central nervous system damage. In cerebral ischemia, for example, CsA has been shown to induce neural tissue protection and neurological improvement [20]. The beneficial effect of CsA has also been explored following spinal cord injury [11]; in such case CsA was able to decrease lipid peroxidation. In our study, we have documented a potentially beneficial effect of CsA on increasing immune activity when administered at minimal and consecutive doses, may be affecting Tregs. Thus, it is important to understand and investigate such differences in order to accurately expand new potential applications in addition to the traditional uses in inhibiting transplant rejection and modulating autoimmunity for this therapeutic agent.

## References

[1] St Clair E.W., Turka L.A., Saxon A., Matthews J.B., Sayegh M.H., Eisenbarth G.S., Bluestone J. (2007). New reagents on the horizon for immune tolerance. Annu. Rev. Med..

[2] Salama A.D., Remuzzi G., Harmon W.E., Sayegh M.H. (2001). Challenges to achieving clinical transplantation tolerance. J. Clin. Invest..

[3] Mellor A.L., Munn D.H. (2000). Immunology at the maternal-fetal interface: lessons for T cell tolerance and suppression. Annu. Rev. Immunol..

[4] Castellino F., Germain R.N. (2006). Cooperation between CD4+ and CD8+ T cells: when, where, and how. Annu. Rev. Immunol..

[5] Cosimi A.B., Sachs D.H. (2004). Mixed chimerism and transplantation tolerance. Transplantation.

[6] Sherman L.A., Chattopadhyay S. (1993). The molecular basis of allorecognition. Annu. Rev. Immunol..

[7] Yamada A., Salama A.D., Sayegh M.H. (2002). The role of novel T cell costimulatory pathways in autoimmunity and transplantation. J. Am. Soc. Nephrol..

[8] Rosenberg A.S., Singer A. (1992). Cellular basis of skin allograft rejection: an *in vivo* model of immune-mediated tissue destruction. Annu. Rev. Immunol..

[9] Krensky A.M., Weiss A., Crabtree G., Davis M.M., Parham P. (1990). T-lymphocyte-antigen interactions in transplant rejection. N. Engl. J. Med..

[10] Bretscher P.A., Havele C. (1992). Cyclosporin A can switch the immune response induced by antigen from a humoral to a cell-mediated mode. Eur. J. Immunol..

[11] Diaz-Ruiz A., Vergara P., Perez-Severiano F., Segovia J., Guizar-Sahagun G., Ibarra A., Rios C. (2004). Cyclosporin-A inhibits inducible nitric oxide synthase activity and expression after spinal cord injury in rats. Neurosci. Lett..

[12] MacLeod H., Goodwin D.G., Damphousse C., Lonie E., Xu X., Collins M., Nickerson-Nutter C.L. (2010). The DTH effector response and IL-2 are unaffected by cyclosporine A in autoimmune B6D2F1 mice. Cell Immunol..

[13] Sigal N.H., Dumont F.J. (1992). Cyclosporin A, FK-506, and rapamycin: pharmacologic probes of lymphocyte signal transduction. Annu. Rev. Immunol..

[14] Shevach E.M. (1985). The effects of cyclosporin A on the immune system. Annu. Rev. Immunol..

[15] Bunjes D., Hardt C., Rollinghoff M., Wagner H. (1981). Cyclosporin A mediates immunosuppression of primary cytotoxic T cell responses by impairing the release of interleukin 1 and interleukin 2. Eur. J. Immunol..

[16] Kronke M., Leonard W.J., Depper J.M., Arya S.K., Wong-Staal F., Gallo R.C., Waldmann T.A., Greene W.C. (1984). Cyclosporin A inhibits T-cell growth factor gene expression at the level of mRNA transcription. Proc. Natl. Acad. Sci. USA.

[17] Harding M.W., Handschumacher R.E. (1988). Cyclophilin, a primary molecular target for cyclosporine. Structural and functional implications. Transplantation.

[18] Ryffel B. (1989). Cyclosporine (Sandimmune) and wound healing. Urol. Res..

[19] Hess A.D. (1993). Mechanisms of action of cyclosporine: Considerations for the treatment of autoimmune diseases. Clin. Immunol. Immunopathol..

[20] Vine W., Bowers L.D. (1987). Cyclosporine: Structure, pharmacokinetics, and therapeutic drug monitoring. Crit. Rev. Clin. Lab Sci..

[21] Pores-Fernando A.T., Gaur S., Doyon M.Y., Zweifach A. (2009). Calcineurin-dependent lytic granule exocytosis in NK-92 natural killer cells. Cell. Immunol..

[22] Bocian K., Borysowski J., Wierzbicki P., Wyzgal J., Klosowska D., Bialoszewska A., Paczek L., Gorski A., Korczak-Kowalska G. (2010). Rapamycin, unlike cyclosporine A, enhances suppressive functions of *in vitro*-induced CD4+CD25+ Tregs. Nephrol. Dial. Transplant..

[23] Miroux C., Morales O., Carpentier A., Dharancy S., Conti F., Boleslowski E., Podevin P., Auriault C., Pancre V., Delhem N. (2009). Inhibitory effects of cyclosporine on human regulatory T cells *in vitro*. Transplant. Proc..

[24] Ciesek S., Ringe B.P., Strassburg C.P., Klempnauer J., Manns M.P., Wedemeyer H., Becker T. (2005). Effects of cyclosporine on human dendritic cell subsets. Transplant. Proc..

[25] Veltman J.D., Lambers M.E., van Nimwegen M., de Jong S., Hendriks R.W., Hoogsteden H.C., Aerts J.G., Hegmans J.P. (2010). Low-dose cyclophosphamide synergizes with dendritic cell-based immunotherapy in antitumor activity. J. Biomed. Biotechnol..

[26] Meloni F., Morosini M., Solari N., Bini F., Vitulo P., Arbustini E., Pellegrini C., Fietta A.M. (2006). Peripheral CD4+ CD25+ Treg cell expansion in lung transplant recipients is not affected by calcineurin inhibitors. Int. Immunopharmacol..

[27] Kasaian M.T., Biron C.A. (1990). Cyclosporin A inhibition of interleukin 2 gene expression, but not natural killer cell proliferation, after interferon induction *in vivo*. J. Exp. Med..

[28] Malard F., Szydlo R.M., Brissot E., Chevallier P., Guillaume T., Delaunay J., Ayari S., Dubruille V., Le Gouill S., Mahe B., Gastinne T., Blin N., Saulquin B., Harousseau J.L., Moreau P., Mohty M. (2010). Impact of cyclosporine-A concentration on the incidence of severe acute graft-versus-host disease after allogeneic stem cell transplantation. Biol. Blood Marrow Transplant..

[29] Behforouz N.C., Wenger C.D., Mathison B.A. (1986). Prophylactic treatment of BALB/c mice with cyclosporine A and its analog B-5-49 enhances resistance to Leishmania major. J. Immunol..

[30] Strom T.B. (2004). Is transplantation tolerable?. J. Clin. Invest..

